# Annotations

**Published:** 1891-03-28

**Authors:** 


### Annotations.

**The Royal Free Hospital and Medical Women.**

We publish elsewhere a letter from Mrs. Garrett Anderson, M.D., in which she seeks to modify certain statements of ours made on evidence given by her before the Lords' Commission. Mrs. Garrett Anderson had said in effect that though the Royal Free Hospital gave many facilities for the education of medical women, it had twice declined to help them in the matter of midwifery training. Upon this we made some rather severe comments, which we need not here repeat. Mrs. Garrett Anderson's letter speaks for itself, and shows that though the managers of the Royal Free Hospital had not been able to provide instruction in midwifery for women, they had provided for them all the facilities that were in their power. Not only had they done this, but they had done it freely, without any pressure from public opinion; and in fact long before general opinion had encouraged them to take any such steps. We gladly emphasise Mrs. Garrett Anderson's corrections. Not only so, but through the courtesy of the hospital authorities we are enabled to add one or two facts which put the hospital managers in quite a new position with regard to the education of medical women. The real state of things has been this, that very early in the medical women's movement the managers of the Royal Free Hospital turned a favourable ear to lady students and did their utmost to provide a medical school for them. It was the wish of the managers to give the ladies full opportunities for learning the practice of midwifery, but this wish was frustrated by certain practical difficulties which arose, and which could not be surmounted. Those difficulties were not all on the side of the hospital managers; the ladies themselves were responsible for some of them, and the medical staff for others. The question of payment also presented an obstacle. The Royal Free Hospital has prided itself on being true to its name. It was founded as a free hospital, and it has always insisted upon preserving its free character. The question of payments either for midwifery or for other work it has refused even to entertain. Perhaps our readers will accept our assurances, based as they are on new knowledge, and taken together with Mrs. Garrett Anderson's letter, that the managers of the Royal Free Hospital not only did not deserve the strictures which we wrote upon them, but that on the contrary they have deserved and do deserve the thanks of all working women and of all men who desire for women freedom to work.

**A Crisis in Child Labour.**

There is a proposal now before the House of Commons which is astonishing in its daring and evil significance. Had we been asked beforehand if we could believe that there was a single member in the whole British House of Commons who would have the hardihood to bring forward such a proposal we should have said, "No, we did not believe it." But there is such a proposal before the House; and there is actually some fear in well-informed quarters that it may become law. Hitherto it has been necessary before children could be employed in factories and workshops that they should be medically "certified" as being fit for such employment. The proposal before the House of Commons is that such certification shall no longer be held necessary, and that the parents or guardians of children and their employers shall be entitled to the free exercise of their own discretion in the matter. A doctor, of course, needs no evidence but his own experience to convince him of the stupidity, cruelty, and wickedness of such a proposal. Non-medical readers, however, have not the doctor's knowledge of the facts, and, therefore, we furnish them with two or three examples of cases which show the urgent necessity for medical "certification" of fitness for child labour. One certifying surgeon, recording two cases, says: "The first, the son of an employer, was suffering from acute curvature of the spine, the result of diseased bone, the disease still being in an active state, there being also a discharging sinus or abscess. The second was of a somewhat similar nature, a boy suffering from caries of the spine, under treatment at a neighbouring infirmary, and at the time of being presented was encased in a plaster of Paris jacket." Another certifying surgeon says: "I had to visit one of the finest and best-appointed factories in England, and there was presented to me a child ten years old to be passed as a half-timer. I observed that he limped as he walked, and I asked the cause He replied that his foot had been scalded. 'How long ago?' 'A tidy time.' On examining the foot I found two deep, inflamed, and painful ulcers, which must have existed for some weeks. I refused to pass the boy. Here was a case where either the cruel apathy or the greed of the parents had produced neglect of the poor child's suffering. But he was actually compelled to attempt to work; and this he would have had to do even in the factory of one of the most humane employers of labour in this country but for the veto of the certifying surgeon." A third certifying surgeon states that he himself has refused certificates in no less than one hundred and ninety-eight cases. In each of these instances the child, by the ignorance or avarice of its parents, would have been put to labour for which it was totally unfit. No fewer than 66 of the certificates were refused because the children were too young, 84 because they were diseased or deformed or too feeble and frail, 35 for ailments of the eyes, and 13 for various forms of heart disease. We do not pretend for a moment to enter into the pros and cons of the case. There are, indeed, no pros and cons about the matter. It is a question which does not admit of argument. We protect horses that are unfit for work, and in case of need we order them to be killed. Hitherto we have protected children. If it has come to this, that parents or the country can no longer support children who are so diseased or so feeble as not to be able to work, then let us, rather than killing them by the slow torture of factory or workshop labour, ask Dr. Richardson to provide us with a lethal chamber in which they may be quietly and painlessly disposed of. The present writer, if he were a member of the House of Commons, would much rather vote for the humane extinction of child life than for its misery and suffering by daily torture. It makes one almost despair of mankind when one sees educated Englishmen in considerable numbers constantly endeavouring to hark back to the ethics of the wolf and the crocodile.

**"Chronic" Students.**

At every hospital and medical school there are young men who have failed repeatedly to pass qualifying examinations, but who still hang on in the medical classes and continue to go up to each successive examination in the hope that by some rare stroke of good fortune they will sooner or later "scrape through." Many of these men lead very unhappy lives, and are the cause of much anxious care to their parents and other members of their families. One of those unhappy men committed suicide a few days ago. Before his death he wrote to one of his relations. "Mine," he said, "has been a sad life, and a wasted career; still, I hope it has not been all bad." These are the notes that chiefly distinguish the life of the chronic student's history. It is a "sad life," and it is mostly a "wasted career." It strikes us that some effort should be made by the authorities of the medical schools to put an end to the circumstances which make the "chronic student" possible. There is no intention here of putting forward a cut and dried plan; the object is to call attention to the matter, and to suggest that it is discreditable to medical schools to have chronic students hanging about. The writer has known young men of respectable families who have been students for seven, eight, nine, ten, and in one case thirteen or fourteen years. Of course, most of them have been ruined men: their mental and moral fibre have been entirely destroyed. The cost of this destructive process to their parents has been enormous. But why, then, do those parents continue to supply their sons with money? asks the uninstructed reader. The money is supplied because the son tells the father at each successive examination that he is sure to pass this time. The father not unnaturally thinks that as the son has been so many years at medical work, he is more likely to get through the examinations and to succeed in the medical profession than he would be to make a successful career by beginning at the beginning of some other calling; and so he keeps on throwing fresh money into a bottomless pit. The medical schools, we say, owe a duty to such a parent. They should at least let him know where his son stands, and what are the prospects of his ever entering the medical profession. In the interests of the larger public, as well as of the parents of chronic students, the schools should prescribe a maximum limit, as they already prescribe a minimum limit of medical study. This may seem a somewhat unpractical proposal. We have not space now to argue it at length; but we are convinced of the urgency of the evils here considered, and of the extreme desirableness of making some attempt to put an end to them.

